# The emerging role of the small intestinal microbiota in human health and disease

**DOI:** 10.1080/19490976.2023.2201155

**Published:** 2023-04-19

**Authors:** Renate A. A. A. Ruigrok, Rinse K. Weersma, Arnau Vich Vila

**Affiliations:** a Department of Gastroenterology and Hepatology, University Medical Centre Groningen, Groningen, The Netherlands; b Department of Genetics, University Medical Centre Groningen, Groningen, The Netherlands

**Keywords:** small Intestine, microbiota, microbiome, metabolism, immunity, human health

## Abstract

The human gut microbiota continues to demonstrate its importance in human health and disease, largely owing to the countless number of studies investigating the fecal microbiota. Underrepresented in these studies, however, is the role played by microbial communities found in the small intestine, which, given the essential function of the small intestine in nutrient absorption, host metabolism, and immunity, is likely highly relevant. This review provides an overview of the methods used to study the microbiota composition and dynamics along different sections of the small intestine. Furthermore, it explores the role of the microbiota in facilitating the small intestine in its physiological functions and discusses how disruption of the microbial equilibrium can influence disease development. The evidence suggests that the small intestinal microbiota is an important regulator of human health and its characterization has the potential to greatly advance gut microbiome research and the development of novel disease diagnostics and therapeutics.

## Introduction

Trillions of bacteria, fungi, viruses, and archaea take residence in our gastrointestinal (GI) tract. These gut-based microorganisms, better known as the gut microbiota, are crucial for human health and perturbations in their preferred assembly have been associated with an extensive range of pathologies.^1^ It is therefore of no surprise that the gut microbiota continues to command the attention of both scientific researchers and the health industry alike.

Although referred to broadly as the gut microbiota, the human intestine harbors distinct communities of microbes – microbial niches – along its length.^2^ With more and more research being conducted, it is becoming increasingly apparent that these microbial niches establish unique interactions with the host and perform different functions within the body. This is significant, considering that the majority of studies investigating the gut microbiota are based on fecal samples – which more accurately represent the distal intestinal (colonic) microbiota – leaving the functions performed by the microbes in the proximal parts of the intestinal tract largely unknown. Given that the small intestine (SI) is essential for nutrient absorption from the diet, as well as provides an extensive and favorable site for important immune, metabolic, neural, and endocrine responses to take place, it is highly plausible that its microbiota is equally fundamental.

Evidence is indeed beginning to emerge that the SI microbiota play a key role in human health and disease. This review discusses the challenges and current methods of studying the SI microbiota, what is known about its composition and temporal dynamics, and finally its potential role in host metabolism, immunity, and disease.

## Studying the human SI microbiome

Studying the SI microbiome in humans is challenging due to its poor accessibility and high temporal dynamics. Many studies currently rely on routine endoscopies, intestinal resections, or sudden death victims, to obtain SI samples.^3–9^ These sampling methods, however, have a number of limitations (Table 1). Firstly, endoscopic procedures are invasive, can only be performed by a specialist, and are therefore not easily justified for individuals without GI-related symptoms. Secondly, the lavage treatment that precedes endoscopic procedures may disrupt the endogenous intestinal environment, introducing bias. Thirdly, these methods are prone to contamination from other parts of the intestinal tract during retrieval of the sample. Furthermore, longitudinal samples are difficult to acquire. Lastly, material obtained during these procedures is predominantly mucosal biopsies. Analysis of the mucosa-associated microbiota is limited by 16S rRNA sequencing, which cannot compete with the higher taxonomic and functional resolution achieved with metagenomic shotgun sequencing. Individuals with an ileostomy or ileal pouch-anal anastomosis provide a unique cohort in which to non-invasively and longitudinally sample the SI lumen, circumventing many of the aforementioned problems.^10–12^ However, the limited number of individuals and disease context pose alternative challenges. An altered anatomy and possible exposure of the luminal contents to the external environment are also suggested limitations; however, according to the findings in the recently published study of Yilmaz et al., the intestinal microbial content is not significantly perturbed as a result of an ileostomy.^13^ Additional methods of sampling the SI microbiota include the novel method detailed by Dreskin et al. for simultaneously sampling the proximal luminal and mucosal gut microbiome, without the risk of contamination that is usually associated with regular endoscopies.^14^ Sampling via this method, however, is limited to the proximal SI and is still invasive. Similar restrictions also apply to the 4-lumen catheter with multiple aspiration ports described in the study of Seekatz et al.^15^ Attempts are also being made to develop endoscopic capsules that allow noninvasive, no contamination sampling along the entire GI tract, with some promising results.^16,17^

**Table 1.** Studies sampling the SI microbiota and their methods.

Study cohort	Sampling method	Location in SI	Microbial analysis	Limitations	Main Species Identified	Reference
54-y-old female, previous good health	Mucosal biopsies via colonoscopy and using a Watson intestinal biopsy capsule	Jejunum Distal ileum	16S rRNA gene amplicon sequencing	Prone to contamination Prior lavage treatment Invasive Low taxonomic resolution Small sample size	Jejunum: *Streptococcus* & *Proteobacteria* Distal ileum: *Bacteroidetes* & *Clostridium* clusters XIVa and IV	Wang et al. 2005 [3]
26 patients who underwent emergency surgery requiring colonic resection	Mucosal biopsies following colonic resection	Terminal ileum	16S rRNA gene amplicon sequencing	Low taxonomic resolution Invasive Difficult interpretation	*Bacteroides, Bifidobacteria, Lactobacilli & Enterobacteria*	Ahmed et al. 2007 [4]
3 deceased elderly individuals	Intestinal contents obtained during autopsy	Jejunum Ileum	16S rRNA gene amplicon sequencing	Small sample size Cannot study microbial dynamics	*Streptococcus*, *Lactobacillus*, ‘*Gammaproteobacteria’*, the *Enterococcus* group & the *Bacteroides* group	Hayashi et al. 2005 [5]
5 individuals with an ileostomy & 4 healthy individuals	Ileostomy effluent Intestinal fluid using an intraluminal naso-ileal catheter	(Distal) SI Jejunum, ileum & terminal	GI tract-specific phylogenetic microarray HITChip	Altered GI anatomy (Ileostomy samples) Potential exposure to external environment (Ileostomy samples) Disease context (IBD) (Ileostomy samples) Risk of contamination (naso-ileal catheter) Invasive (naso-ileal catheter)	*Streptococcus, E.coli*, *Clostridium*, high G+C organisms	Zoetendal et al. 2012 [12]
5 participants undergoing colonoscopy	Mucosal biopsies via colonoscopy	Terminal ileum	Metagenomic shotgun sequencing	Small sample size Prior lavage treatment Large number of human reads, low microbial coverage	*B. vulgatus, B. thetaiotaomicron, B. uniformis & B. caccae*	Vaga et al. 2020 [6]
Liver disease patients undergoing esophagogastroduodenoscopy	Duodenal aspirate and biopsies during upper endoscopy – novel technique detailed by the authors	Duodenum	16S rRNA gene amplicon sequencing	Limited to the duodenum Invasive Disease context	*Aspirate: Veillonella, Streptococcus, Prevotella* *Biopsy: Streptococcus, S24–7 (Muribaculaceae), Parabacteroides*	Dreskin et al. 2021 [14]
49 individuals with an ileostomy 9 individuals with an Ileoanal pouch	Ileostomy effluent Fecal sample	SI	Metagenomic shotgun sequencing	Disease context (IBD) Altered GI anatomy (Ileostomy samples) Potential exposure to external environment (Ileostomy samples) Limited sample population	*Streptococcus, Escherichia, Veillonella, Blautia, Actinomyces, Enterococcus, Lactobacillus*	Ruigrok et al. 2021 [10]
27 patients undergoing radical cystectomy	Lab cultivation of samples obtained by rubbing a swab against the ileal mucosa 25 cm from the ileocecal valve	Ileum	MALDI-TOF/Biotyper	Limited to cultivable microbes Disease context may have an influence on the microbial composition	*viridans streptococci, Candida, Actinomyces, Rothia & Lactobacillus species.*	Villmones et al. 2021 [7]
8 fasted healthy individuals	Orally intubation with a four-lumen catheter with multiple aspiration ports – multiple samples	Duodenum Jejunum	16S rRNA gene amplicon sequencing	Invasive Risk of contamination Limited to proximal SI	*Firmicutes, Proteobacteria & Bacteroidetes (duodenum)*	Seekatz et al. 2019 [15]
Ex vivo study on lamb SI	A novel capsule robot designed by the authors: samples mucosa and intestinal contests	SI	/	Still under development	*/*	Rehan et al. 2020 [16]
27 patients undergoing radical cystectomy with urinary diversion	Sample obtained valve by rubbing a swab against the luminal wall about 25 cm proximal to the ileocecal during surgery	Distal ileum	16S rRNA gene amplicon sequencing plus targeted sequencing of alternative genes when necessary for higher taxonomic resolution	Disease context may have an influence on the microbial composition	*Streptococcus, Granulicatella, Actinomyces, Solobacterium, Rothia, Gemella & TM7(G-1)*	Villmones et al. 2018 [8]
6 patients undergoing gastric bypass surgery	Sample obtained valve by rubbing a swab against the luminal wall during surgery	Jejunum	16S rRNA gene amplicon sequencing	Disease context may have an influence on the microbial composition	*Streptococcus, Granulicatella, Schaalia odontolytica complex & Gemella*	Villmones et al. 2022 [9]
7 individuals with an ileostomy	Ileostomy effluent	SI	HITChip based on SSU rRNA gene sequences	Disease context (IBD) Altered GI anatomy Potential exposure to external environment Limited sample population	*Streptococcus, Veillonella & Clostridium cluster I*	Booijink et al. 2010 [11]
30 individuals with an ileostomy	Ileostomy effluent	SI	16S rRNA gene amplicon sequencing	Disease context (colorectal cancer) Altered GI anatomy Potential exposure to external environment Limited sample population	*Lactobacillus, Clostridium, Streptococcus*, *Enterococcus, & Veillonella*	Yilmaz et al. 2022 [13]

Abbreviations: SI, small intestine.

NB: Includes studies that also sampled the large intestine; however, only the methods used to study the SI and the corresponding results are detailed.

In addition to sampling, efforts have been made to model the SI both *in vitro* and *in vivo*. A potentially promising *in vitro* model for the human ileum and its microbiota is the single-stage fermenter developed by Stolaki et al.^18^ *In vivo* model studies have largely involved mice. Although differences between mouse and human GI tract and microbial composition are known – such as tract length in relation to the size of the species^19^ - these studies have allowed for functional exploration into the role of the SI microbiota in both healthy and disease-specific contexts. Future studies could also consider using piglet models given they have a more anatomically and physiologically similar GI tract to humans than mice.^20^

Overall, while the importance of the SI microbiota is gaining support and progress is being made to study its composition and function, there is still a need to develop uniform techniques that accurately model its dynamics.

## The composition and temporal dynamics of the human SI microbiota

Despite the differences in study populations, sampling techniques, and analytic methods that complicate the process of characterizing the human SI microbiota, studies appear to agree on the main characteristics of this microbial ecosystem.

The microbial density in the SI microbiota is estimated at 10^3^ - 10^8^ cells/g, with an increasing gradient going from a low density in the duodenum (10^3^) to a high density in the terminal ileum (10^8^) which is still approximately 4-fold lower than in the colon.^21^ This increasing gradient reflects the more favorable conditions – lower acidity and lower concentrations of digestive enzymes and pancreatic, gastric, and bile acids – found in the terminal ileum compared with the duodenum and jejunum.^2,22^ The microbial ecosystem in the SI is also in general less diverse and more dynamic than in the colon^11^; to what extent, it depends on the location along the SI and whether one is describing the luminal content or mucosa-associated microbiota. The mucosal diversity in the ileum and colon, for example, do not differ significantly, whereas when comparing the luminal contents this difference is more pronounced.^23^ Spatial and temporal analysis of the SI microbiota has also shown that there is higher inter- and intra-individual variability in duodenal aspirates compared with those from the jejunum, suggesting that the duodenal microbial community is more dynamic.^15^

The SI microbiota is composed primarily of facultative anaerobic and aerobic bacteria, in particular, belonging to the phyla *Firmicutes, Proteobacteria* and *Actinobacteria*.^3,5,8,10,12,14,15^ This is in contrast to the obligate anaerobes that predominate in the colon.^3,5,12^ At the genus level, bacteria highly abundant in the SI include *Lactobacillus*, *Veillonella*, *Streptococcus*, *Gemella, Actinomyces*, and *E. coli*.^3,5,8,10,12,14,15^ Higher proportions of Bacteroidetes, namely *Prevotella*, and lower proportions of Firmicutes (*Streptococcus*, *Veillonella*, *Gemella*, and *Lactobacillaceae*) are found in the duodenum compared with the jejunum.^15^ Bacterial compositions in the ileum and jejunum appear to be comparable, despite a higher bacterial load in the ileum.^2,8,9^ Only select bacteria, including *Clostridium, Lactobacillus*, and *Enterococcus* species, are able to penetrate, or attach to, the mucus layer at the epithelium and use the mucus as an energy source, explaining some of the differences seen between the bacterial communities in the lumen versus the mucosa.^24,25^

The composition of the SI microbiota is also influenced by external factors. Two important factors are smoking and proton pump inhibitor (PPI) therapy.^26,27^ Smoking has been shown to disrupt both the duodenal mucosa-associated and luminal microbiota.^26,27^ In the lumen, the abundance of *Prevotellaceae*, *Neisseriaceae*, and *Porphyromonadaceae* is reduced in smokers, whereas *Enterobacteriaceae* and *Lactobacillaceae* abundances are increased. Smoking cessation eventually restores microbial composition to the extent that it more closely resembles that of a never-smoker. PPIs are a commonly prescribed drug for the prevention or treatment of gastric-acid related diseases, such as stomach ulcers and reflux complaints. PPIs inhibit gastric acid secretion, raising stomach pH and allowing certain microbes to thrive and colonize more distal sections of the GI tract. In the study of Lim et al., PPI use was shown to induce duodenal microbiota dysbiosis, characterized by an increase in *Akkermansia muciniphila* and *Porphyromonas endodontalis* and a decrease in *Enterococcaceae*, *Coprococcus*, *Enterobacteriaceae*, and *Synergistes* species.^27^

Rapid changes in the nutrient availability also acts as a factor shaping the microbiota composition along the small intestine. Using luminal stoma (ileo- and colostomy) contents from 114 patients with IBD or colorectal cancer, Yilmaz and colleagues demonstrated dynamic changes in the ileostomy-derived bacterial biomass and strain profiles following dietary intake,which was in contrast to the more stable microbiota of colostomy-derived samples.^13^

Lastly, it should be noted that belonging to SI microbiota are also viruses, archaea, and fungi. Although almost completely unexplored in the context of the SI, these microbes are increasingly being studied in the context of the fecal microbiome and are proving highly important for not only shaping the microbiota but also maintaining health and driving disease.^28,29^ Using ileostomy fluid and fresh colon resections from non-IBD and IBD patients to study the protective or pro-inflammatory potential of the viruses residing in the gut, the study of Adiliaghdam and colleagues gives us a first glimpse into the SI virome.^30^ The results revealed that eukaryotic viral families present in the SI include Anelloviridae, Papillomaviridae, Picornaviridae, and Virgaviridae, and bacteriophage families include Caudovirales, Siphoviridae, Myoviridae, and Podoviridae. Additional studies, however, are necessary to confirm these findings and define, if existing, a core SI virome. Given the paucity of studies describing the non-bacterial component of the SI microbiota, this review will henceforth continue to focus on the SI bacteriome.

## The SI microbiota and host metabolic regulation

**Figure 1.**
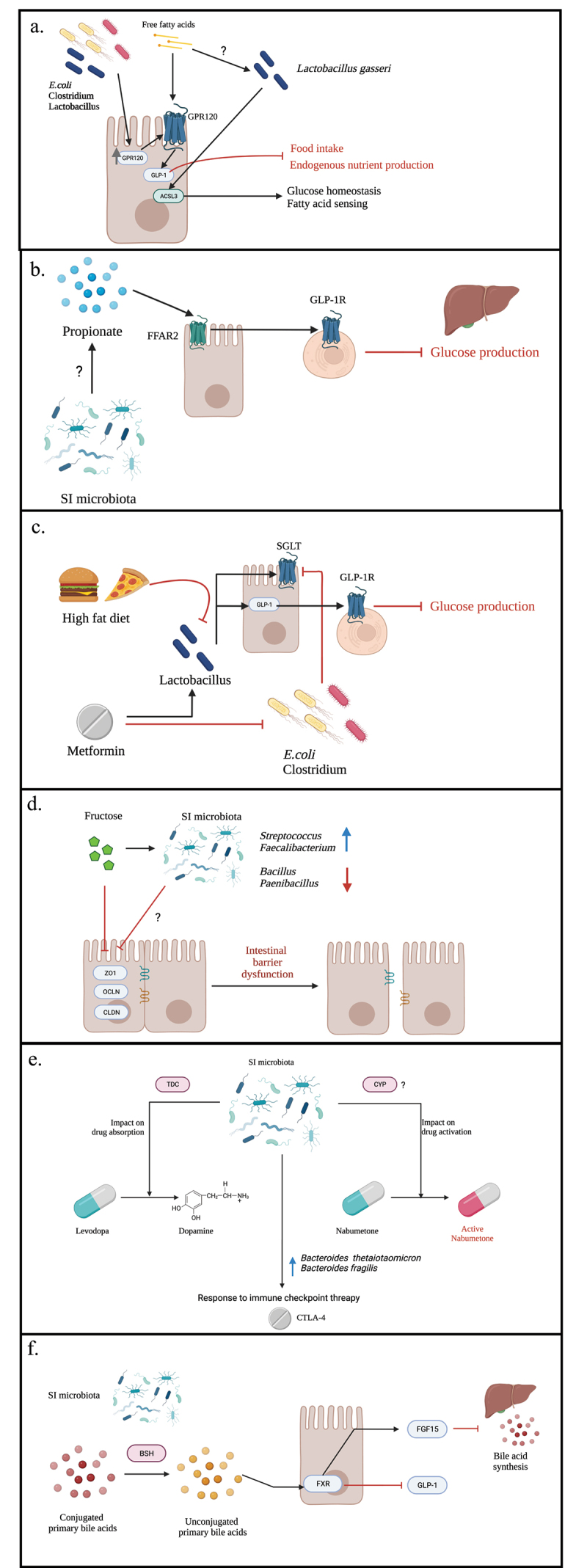
Interactions of small intestinal microbes and host metabolism.

Note: A schematic overview of the concepts discussed throughout this review regarding the role of the small intestinal microbiota in host metabolism. **(a-c) nutrient sensing and energy homeostasis: a)** SI bacteria can influence energy homeostasis and nutrient sensing through upregulation of GPR120 expression which, upon activation by free fatty acids, stimulates GLP-1 secretion. GLP-1 secretion in turn leads to decreased energy intake and endogenous nutrient production. SI bacteria may also facilitate fatty acid nutrient sensing and glucose homeostasis through increased ACSL3 protein expression and downstream signaling leading to decreased glucose production. **b)** It is proposed that SI bacteria can produce short-chain fatty acids (represented in the figure by propionate) which, via an FFAR2/GLP-1R dependent pathway, regulates glucose metabolism. **c)**A high fat diet and metformin modulate the SI microbiota in opposing ways which influence glucose metabolism. **(d) fructose metabolism:** excessive fructose intake is associated with reduced tight junction gene expression (ZO1, OLCN, CLDN) possibly mediated by (fructose-induced) SI microbial dysbiosis, leading to intestinal barrier dysfunction. **(e) drug interactions:**SI microbes are involved in the bioavailability of certain prescription drugs, via endogenous enzyme activity (TDC and CYP, respectively). Similarly, SI bacterial abundances are associated with the efficacy of anti-tumor treatments, namely CTLA-4 immunotherapy. **(f) bile acid metabolism:**SI microbes regulate bile acid pools in the small intestine via BSH enzyme activity, which in turn regulates bile acid synthesis and host/lipid metabolism. *Figure created using BioRender (biorender.com)*

In addition to being the primary site of nutrient and energy absorption from the diet, the SI performs a number of other functions that are critical for host metabolism. In line with this, studies exploring the potential function of the SI microbiota using computational analyses have revealed an enrichment of bacterial pathways associated with energy and simple carbohydrate metabolism.^10,12^ In this section, we discuss the role of the SI in regulating host metabolism and the emerging evidence that places the SI microbiota in this role (Figure 1).

### Nutrient sensing and glucose/energy homeostasis

The SI has the ability to sense nutrients and trigger both local and systemic, hormonal, and neurological responses in the body that regulate energy and glucose homeostasis.^31^ Distributed throughout the intestinal epithelium – but of specific importance, here, in the SI – are hormone-secreting enteroendocrine cells (EECs)^32^ which, upon exposure to nutrients, secrete a variety of gut peptides including the hormones glucagon-like peptide 1 (GLP-1) and cholecystokinin (CCK) .^33,34^ These secretory products in turn act in an endocrine or paracrine fashion via the gut–brain metabolic axis and/or portal vagal-brain axis to prevent postprandial excess energy by suppressing food intake and endogenous nutrient production.^31,35–37^

Tahmasebi and colleagues were able to show that propionate infusion into the ileum of rats activates ileal mucosa-located free fatty acid receptor 2 (FFAR2).^38^ FFAR2 activation in turn triggers a negative feedback signal that, via a GLP-1 receptor-dependent neural network, results in reduced hepatic glucose production. Propionate is a short-chain fatty acid (SCFA) produced by bacterial dietary fiber fermentation. Although SCFAs are primarily synthesized by bacteria in the large intestine,^39^ these results suggest that ileal-derived fatty acids are important for GLP-1 release and glucose homeostasis. In line with this, supernatant from bacteria commonly found in the SI (*Lactobacillus*, *Enterococcus*, *E. coli*) have been demonstrated to increase G protein-coupled receptor 120 (GPR120) expression which, upon stimulation by free fatty acids, promotes secretion of GLP-1.^40^ Furthermore, changes to the upper SI microbiota composition in rats, as a result of a high-fat diet (HFD), were associated with disrupted upper SI fatty acid nutrient sensing and glucose homeostasis, driven by a decrease in long-chain fatty acyl-CoA synthase (ACSL3) expression.^34^ Direct transplantation of upper SI microbiota derived from rats fed a regular chow diet, as well as *L. gasseri* probiotic administration, reverses this phenotype, suggesting that SI bacteria are critical for the ACSL3-dependent regulation of glucoregulatory fatty acid-sensing pathways (Figure 1 a-b).

The role of microbes in SI nutrient sensing is further supported by the study of Bauer et al., investigating the glucose production reducing effect of diabetic medication, metformin.^41^ Metformin had previously been shown to increase upper SI GLP-1 secretion, stimulate upper SI sodium glucose cotransporter-1 (SGLT1) expression which regulates GLP-1 secretion, and lower glucose production through a GLP-1 R dependent neuronal axis, when infused into the upper SI.^42–45^ Furthermore, metformin intake was associated with alterations to the distal gut microbiota^46^, leading Bauer and colleagues to investigate whether changes to the upper SI microbiota also play a role in the glucoregulatory effects of metformin treatment.^41^ First, they confirmed that rats fed a HFD for as little as 3 days had disrupted upper SI glucose sensing, characterised by reduced SGLT1 expression and GLP-1 secretion. They then showed that an upper SI microbiota transplant from metformin-treated donor rats, leading to an increase in *Lactobacillus salivarius* and a decrease in *Clostridium* and *E. coli* in the upper SI, was able to restore the defective glucose sensing in HFD fed rats. Taken together, these results suggest that the glucoregulatory effects of metformin are partially driven by compositional changes in the upper SI microbiota, including reversal of the HFD-induced decrease in *Lactobacillus* species (Figure 1c).

### Circadian rhythms and metabolism

Circadian rhythms are the physical, mental, and behavioral changes occurring within an organism that continuously cycles over a 24-hour period.^47^ Circadian rhythms are driven by a 24-hour internal clock – the circadian clock – found in almost all cells of the body, which is in turn driven by the light-regulated, ‘central’ circadian clock located in the brain.^48^ ‘Peripheral’ circadian clocks can also be influenced by factors other than the central clock, such as the time of food consumption, which regulates liver and intestinal circadian rhythms.^49,50^ Circadian rhythms are important for maintaining cellular and organ function, and dysregulation of the rhythms can lead to organ dysfunction. The link between circadian rhythms and host metabolism is well acknowledged^47^ and the SI and its microbiota appear to play an important role here.^51^ Previous studies had shown that diurnal fluctuations in intestinal microbial composition are necessary to the entrain peripheral circadian clocks that, in turn, maintain host metabolic homeostasis by inducing diurnal expression of hepatic and intestinal metabolic regulators of glucose, cholesterol, and fatty acid metabolism.^48,52–54^ However, these findings are based primarily on fecal samples, largely representing the large intestine. Dantas Machado and colleagues therefore focused on the process of peripheral circadian rhythm entrainment in the ileum.^51^ Here, they demonstrated that diurnal oscillations in ileal microbial composition and host ileal transcriptome exist, and both are disrupted in diet-induced obese mice, leading to perturbed peripheral circadian rhythms and dysmetabolism. Interestingly, time-restricted feeding was able to restore the ileal diurnal microbial dynamics, increase GLP-1 production and influence bile acid pools and signaling, suggesting that microbial dynamics in the SI are important for regulating host metabolism, and dietary/feeding patterns can significantly influence these dynamics.

### Bile acid metabolism

The central role of the SI in bile acid metabolism is another important aspect of the SI’s regulatory function of host metabolism. Bile acids represent a key group of signaling molecules in the gut that primarily facilitate absorption of lipids and fat-soluble nutrients in the SI, but also regulate lipoprotein, glucose, and energy metabolism.^55,56^ Bile acids carry out their signaling functions by binding to nuclear receptor, farnesoid X receptor (FXR) and membrane-bound G protein-coupled bile acid receptor (TGR5). Both receptors are highly expressed in the ileum, where 90% of bile acids get reabsorbed. Constitutive bile acid-mediated activation of intestinal FXR has been shown to worsen obesity and increase insulin resistance and fatty liver.^57–59^ In the mouse study conducted by Jiang et al., FXR signaling inhibition reversed the metabolic dysfunctions associated with a high-fat diet and a genetic predisposition for metabolic related diseases, including obesity, insulin resistance, and fatty liver.^60^ Moreover, FXR signaling was enriched in the distal ileal mucosa of obese individuals, and mRNA levels of FXR signaling pathway molecules: *fxr*, small heterodimer partner (*shp*) and fibroblast growth factor 19 (*fgf19*), correlated with body mass index in these same individuals. FXR activation inhibits GLP-1 transcription and secretion by intestinal epithelial L-cells^61^, suggesting the beneficial metabolic effects of FXR inhibition involve increased intestinal GLP-1 production. In line with this, are the findings reported by Thomas and colleagues from their work in obese mice. TGR5 signaling was shown to stimulate intestinal GLP-1 release, which resulted in improved liver and pancreatic function with increased glucose tolerance in obese mice.^62^ These results emphasize the role of bile acid signaling in the intestinal regulation of energy and glucose homeostasis.

The gut microbiota is a key regulator of bile acid metabolism and signaling. Primary bile acids (PBAs), namely cholic acid (CA) and chenodeoxycholic acid (CDCA) in humans, are produced in the liver.^56,63^ PBAs are subsequently conjugated with glycine or taurine – making them water soluble – and secreted into the bile where they are either stored in the gallbladder or directly released into the SI for lipid and fat-soluble nutrient absorption. Once in the SI, the fate of PBAs is variable. Some PBAs are passively absorbed in the SI in their conjugated form, others are first deconjugated by SI bacteria before being passively reabsorbed, the majority are actively reabsorbed in the terminal ileum and approximately 5% continue on into the colon where they are converted by colonic bacteria into secondary bile acids (SBAs) and excreted in the feces. A small proportion of SBAs are also reabsorbed in the colon. Intestinal microbes are therefore critical for modulating host bile acid pools, which has an influence on host physiology. More detail on this microbial-dependent regulation of bile acid pools can be found in the review of Winston and Theriot^63^. The effect of SI bacteria on bile acid signaling and host metabolism is discussed further in this review in the context of metabolic diseases in the section ‘The SI microbiota and disease’.

### Fructose metabolism

A more recently discovered function of the SI in fructose metabolism also warrants attention considering the strong correlation between high fructose intake and metabolic-related diseases such as obesity, type 2 diabetes, nonalcoholic fatty liver disease, kidney dysfunction, and cardiovascular disease.^64^ The conversion of fructose to glucose was previously assumed to takes place in the liver, on the basis that high levels of fructose catabolic enzymes are expressed in the liver, and the liver is highly sensitive to fructose leading to fatty liver disease.^64,65^ Jang and colleagues, however, revealed that the SI is in fact responsible for the conversion of fructose to glucose.^66^ This is hypothesized to play a protective role against fructose toxicity in the liver, provided fructose intake does not exceed the SI’s metabolic capacity. If fructose intake exceeds this threshold, fructose becomes available for intestinal bacteria, which, at least in the colon, can convert fructose into potentially hepatotoxic metabolites. Excess fructose intake may also drive intestinal barrier dysfunction, as a result of SI microbial dysbiosis.^20^ Fructose supplementation in piglets led to reduced ileal expression of three main tight junction genes, *ZO1*, *OCLN*, and *CLDN*, which correlated with decreased *Bacillus* and *Paenibacillus* (aerobes) and increased *Streptococcus* and *Faecalibacterium* (anaerobes) relative abundances in the ileum.^20^ Investigating the role of SI bacteria in fructose metabolism could therefore have significant implications for metabolic health (Figure 1d).

### Drug bioavailability and response

The SI is also an important site for drug absorption, having a significant influence on drug efficacy. Bacteria in the gut can also metabolize drugs; however, studies have mainly focused on the metabolism occurring in the colon and, thus, the proportion of drugs that are not absorbed by the host.^67^ Bacteria in the SI, on the other hand, have the opportunity to influence the bioavailability of orally administered drugs by modulating the drug before the host has had a chance to absorb it. In the study performed by van Kessel and colleagues, fecal samples from patients with Parkinson’s disease (PD) and rats were used to investigate the possible modulation of levodopa by gut microbes.^68^ Levodopa, in combination with a decarboxylase inhibitor, is the primary treatment for PD – a degenerative disorder of the central nervous system. Treatment, however, is not always successful with a number of patients either not responding or having to increase their dosage regimen due to a decrease in treatment response over time. Given that the proximal SI (jejunum) is the primary site for levodopa absorption, and levodopa closely resembles the substrate of bacterial tyrosine decarboxylases (TDC),^69–71^ jejunal bacteria were hypothesized to mediate this reduced response to treatment. Van Kessel and colleagues observed that proximal SI bacteria, via tyrosine decarboxylase activity, were indeed able to efficiently convert levodopa to dopamine – the active form that is unable to cross the blood–brain barrier and exert its disease-modulating effects. The results implicate jejunal bacteria in the bioavailability of levodopa, providing a possible explanation as to why differences in the efficacy of levodopa are seen between patients with PD. Similarly, gut microbes are also linked to the metabolism of the nonsteroidal anti-inflammatory drug, nabumetone.^72^ Germ-free (GF) mice, compared with specific pathogen-free mice, exhibit increased small intestinal expression of drug metabolizing, cytochrome P450 (CYP) enzymes following oral administration of nabumetone. Cytochromes P450 convert nabumetone to its active form, highlighting the importance of gut microbes, plausibly specifically those in the SI, in regulating the effects of (nabumetone) drug treatment (Figure 1e).

In addition to drug bioavailability, the microbiota is related to drug response possibly via an interaction with the immune system. Antibodies targeting the immune checkpoint receptor protein and negative regulator of T cell activation, CTLA-4, have been successfully used to treat various cancers and improve survival outcomes, especially in the case of melanoma.^73^ The tumor fighting effect of CTLA-4 antibodies was studied in relation to gut microbes by Vétizou et al.^74^ Following injection of the CTLA-4 antibodies in wild-type mice, Vétizou and colleagues observed an increase in the relative abundance of *Bacteroides thetaiotaomicron* and *Bacteroides fragilis* at the SI mucosa, which was positively associated with CTLA-4 efficacy. These antitumor effects were absent or reduced in GF mice and mice treated with antibiotics, which were restored upon recolonization with *B. thetaiotaomicron* and *B. fragilis*. The role of SI microbes, therefore, also likely extends to the immunostimulatory effects of cancer immunotherapy, which if investigated further could tremendously enhance cancer therapy (Figure 1e).

## The SI microbiota and immunity

The gut-associated lymphoid tissue (GALT) constitutes approximately 70% of the entire immune system in the human body, and the largest component is located in the SI.^75^ This immune tissue is not only critical for the maintenance of intestinal homeostasis but also protects the body against external factors that are able to penetrate the intestinal mucus barrier. Key to its role in maintaining intestinal immune homeostasis, GALT must sustain an equilibrium between, on the one hand, tolerance for diet-derived antigens and commensal microbes and, on the other hand, immunity toward pathogenic stimuli.^76^ Failure to maintain this equilibrium underlies the development of intestinal and extraintestinal immune-related disorders, such as type 1 diabetes, celiac disease, and inflammatory bowel disease (IBD). GALT has also more recently been linked to the development and organization of the enteric nervous system, which is closely linked to the central nervous system.^77^ Intestinal muscularis macrophages in particular have been shown to play a significant role here. Mice depleted of muscularis macrophages show gut dysmotility and a less-organized enteric nervous system.

GALT can be divided into three distinct components distributed within the different layers of the intestinal wall: diffuse lymphoid tissue within the lamina propria, isolated lymphocytes embedded within the epithelium (intraepithelial lymphocytes, IELs) and organized lymphoid follicles such as Peyer’s patches in the ileum.^78^ The diffuse lymphoid tissue is mainly composed of plasma cells, but also contains T lymphocytes (predominantly CD4+) and other innate immune cells. IELs mostly comprise CD8+ T cells and are almost exclusive to the intestinal immune system. Natural killer (NK) cells and NK-T cells are also significantly present in the epithelium. Peyer’s patches are abundant in CD4+ and CD8+ T cells and B cells, and have an overlying epithelium composed of M cells which allow transport of luminal antigens into the patches for an adaptive immune response.^79^ During inflammatory responses, these cells secrete increased levels of chemokines and IgA, which further perpetuates the inflammatory response.

Gut commensals are reliant on the intestinal immune system for their coexistence with the host, and increasing evidence shows that this dependence is mutual; the immune system is equally reliant on gut microbes for its own maturation and regulation.^80^ GF mice exhibit reduced gut lymphoid tissue expansion and present with several immunodeficiencies.^78^ Probiotic treatment with *Lactobacillus kefiri* in mice leads to increased fecal IgA abundances, reduced expression of proinflammatory mediators, and enhanced anti-inflammatory IL-10 release from Peyer’s patches and mesenteric lymph nodes^81^. Furthermore, bacteria-derived metabolites, such as SCFAs, tryptophan, and bile acid derivatives from bile salt hydrolase activity display immunoprotective properties.^82–85^ SCFAs, for example, increase antimicrobial peptide and mucus production and stimulate maturation and expansion of colonic regulatory T cells to reduce local inflammatory responses.^83,85^

Studies implicating SI microbes, specifically, are also emerging.^86^ This is not unexpected given the vast area of immune tissue in the SI alone and the fact that the SI mucus barrier is far less established than in the colon, allowing for closer contact between intestinal epithelial cells and luminal bacteria.^87^ One of the first steps in the intestinal defense cascade is the production and secretion of antimicrobial proteins (AMPs) by intestinal epithelial cells – a feature of innate immunity.^78^ AMP secretion, among other things, regulates bacterial colonization of the mucus, influencing an individual’s likelihood of infection. Expression of multiple AMPs, including regenerating islet-derived protein 3ɣ (REG3G) in the SI epithelium, in mice shows diurnal rhythmicity driven by an ICL3-STAT3 immune signaling pathway.^86^ Regulation of the ICL3-STAT3 immune signaling pathway is in turn dependent on host feeding-regulated rhythmic attachment of segmented filamentous bacteria (SFB) to the SI epithelium. These results indicate that, in addition to the diurnal microbial fluctuations in the SI that regulate host metabolism, rhythmic changes in the SI microbiota also influence diurnal rhythms in intestinal innate immunity that drive the variation in an individual’s susceptibility to infection across the day–night cycle.

Diurnal regulation of immune activity is also important for an adaptive response to repeated delivery of food into the SI. As mentioned earlier, the intestinal immune system must maintain a balance between immunity against pathogenic stimuli and tolerance for nonpathogenic antigens. The continual shifts in nutrient availability in the SI challenge this equilibrium. Resident epithelial cells and IELs must therefore be able to adapt to these changes. SI epithelial cells exhibit diurnal expression of the major histocompatibility complex (MHC) class II complex, which is dependent on host feeding-driven diurnal fluctuations in SI microbes.^88^ This diurnal expression of MHC class II establishes diurnal activation of intraepithelial T cell IL-10+ lymphocytes and thus diurnal secretion of IL-10, which leads to diurnal variation in SI barrier function, namely gut permeability. Such findings highlight the central role of SI microbes in maintaining intestinal tolerance for a highly variable, food-derived antigen burden.

Similarly, in the recent work of Grace Cao and colleagues, where mucosal immunity in the duodenum of mice was explored, *Faecalibaculum rodentium* along with other specific members of the duodenal microbiota was found to regulate duodenal epithelial homeostasis through increasing epithelial cell turnover rate, crypt proliferation, and MHC class II expression.^89^ This was further explained through microbial suppression of enterocyte retinoic acid production, leading to decreased eosinophil populations in the proximal SI. Decreased populations of eosinophils resulted in increased IEL-mediated production of interferon-γ, which stimulated the intra-epithelial cell turnover and MHCII expression.

The presence of immunomodulatory bacteria in the ileum has also been suggested in the context of allergy prevention. Following colonization of GF mice with feces from either healthy or cow’s milk allergy infants, Feehley et al. observed that mice colonized with the feces from healthy infants were protected against anaphylactic responses to cow’s milk allergen.^90^ Further correlation analyses between ileal bacteria and genes upregulated in the ileum identified the Clostridia species, *Anaerostipes caccae*, as a key player in this protective response. Additionally, antigen-specific Th2-dependent antibody and cytokine responses were reduced in mice monocolonised with *A. caccae*. It is therefore possible that *Anaerostipes caccae* induces a specific ileal epithelium gene expression profile, which prevents/attenuates an allergic response to dietary antigens.

Taking these results together, the SI is a key organ where interactions between the microbiota, environmental exposures (e.g., diet and infectious agents), and intestinal immune responses take place to maintain intestinal homeostasis and prevent immune-mediated disease development.

## The SI microbiota and disease

The gut microbiota has been implicated in the pathogenesis of a myriad of diseases, and the mechanisms underlying these roles have been extensively studied and reviewed in relation to the large intestinal microbiota. In this section, the findings and potential mechanisms that explain the role of SI microbiota in the development and treatment of specific disorders will be discussed.

### Functional GI disorders

Functional gastrointestinal disorders (FGIDs), which include functional dyspepsia (FD) and irritable bowel syndrome (IBS), have long been associated with a quantitative increase in bacteria in the SI, known as small intestinal bacterial overgrowth (SIBO).^91^ The mechanisms underlying this pathology, however, remain poorly understood and several studies have since failed to find a correlation between the presence of functional GI symptoms – such as diarrhea, abdominal pain and bloating – and SIBO, questioning the validity of this association.^91–94^ Discrepancies in the association observed between SIBO and the presence of dysbiosis are also present.^91,95,96^ Saffouri and colleagues, for example, failed to demonstrate a correlation between SIBO and duodenal luminal dysbiosis in patients with GI symptoms.^91^ Similarly, Yang et al. reported that SIBO does not correlate with duodenal luminal dysbiosis in patients with diarrhea-predominant subtype IBS (IBS-D); however, it does correlate with duodenal mucosal dysbiosis.^95^ In contrast, Bamba et al. – with the aim of identifying differences in the duodenal microbiome of symptomatic individuals with and without SIBO – found SIBO to be associated with duodenal luminal dysbiosis, characterized by significantly reduced α-diversity and compositional changes including an increase in *Streptococcus spp*. and *Actinomyces spp*. and a decrease in species belonging to the genera *Bacteroides, Blautia*, and *Prevotella*.^96^ These incongruities highlight the limitations of current SIBO diagnostic methods and challenge the significance of a SIBO-positive result, but also emphasize the need for deeper characterization of the SI microbiota in the context of FGIDs to elucidate its role in GI symptom development.

In the study of Zhong et al. involving nine patients with FD, quality of life (QoL), symptom responses to a standardized nutrient challenge and bacterial load – reported as the ratio between copies of bacterial 16S rRNA and human β-actin genes measured using quantitative PCR – were assessed.^97^ Interestingly, bacterial load negatively correlated with reported QoL and positively correlated with the total score of meal-related symptoms following the nutrient challenge, suggesting that bacterial load may be a better predictor of GI symptom development compared to SIBO. Similarly, higher absolute loads of disrupter taxa – bacteria identified as displacing common strict anaerobes in the duodenum – were associated with more severe GI symptoms.^98^ FD has been associated with an enrichment of *Streptococcus*, and a reduction in *Prevotella*, *Veillonella*, and *Actinomyces* species at the duodenal mucosa.^97,99^ Notably, a higher abundance of mucus-associated *Streptococci*, was also observed following PPI use in both patients with FD and healthy controls.^100^ The enrichment of *Streptococcus* bacteria observed at the mucosa in patients with FD may therefore reflect the effect of PPI use, rather than FD itself, on duodenal bacterial composition. The microbial composition at the duodenum mucosa, in the context of FD, is further influenced by the presence of an *H. pylori* infection^101^. Changes to the duodenal luminal microbiota in patients with common FGID symptoms include decreases in *Porphyromonas*, *Prevotella*, and *Fusobacterium* species.^91^ Investigating whether diet-associated increases in FGID symptom burden could be attributed to changes in the SI microbiota, Saffouri and colleagues also performed a short-term dietary intervention pilot study on 16 healthy individuals consuming a baseline high fiber diet.^91^ Participants were placed on a low fiber, high simple-sugar diet for 7 days, and stool and duodenal aspirates were obtained before and after the intervention. 80% of the participants developed GI symptoms during the course of the intervention, which was associated with a concomitant decrease in microbial diversity. Furthermore, there was an inverse relation between SI microbial diversity and SI permeability. A more recent study, conducted by Shanahan and colleagues, on the contrary, found no significant differences in long-term nutrient intake or quality of diet between individuals with FD (*n* = 56) and healthy controls (*n* = 30) nor did they find an association between habitual diet and duodenal mucosa-associated microbiota profiles.^102^ These results suggest that short-term diet-microbe-host interactions are important in driving the development of GI symptoms. How exactly microbes mediate the interaction between host and diet in the pathogenesis of FGIDs is still to be determined. However, in our recent study of the human SI microbiota we found that methane-producing bacterial pathways were significantly underrepresented in the SI when compared with the fecal microbiome.^10^ Methane has been linked to GI motility and constipation-predominant diseases, providing one possible theory implicating SI dysbiosis via increased methane production in GI symptom development.^103,104^

### Obesity and metabolic disorders

As discussed earlier, the SI plays a significant role in maintaining host metabolic homeostasis. Disruption to this state of equilibrium forms the basis of metabolic disorder development, such as obesity, dyslipidemia and type 2 diabetes. In this section, we discuss dyslipidaemia and how the SI microbiota might be involved in driving a hyperlipidaemic state in humans.

Dyslipidemia, or hyperlipidemia, is the presence of excess fat or lipids such as cholesterol and triglycerides in the blood.^105^ Hyperlipidemia is an increasing health problem worldwide, and the complications associated with it, including cardiovascular disease, are devastating. Early treatment is paramount to complication prevention, in return also relieving the increasing pressures health care services are currently facing. GF mice have been shown to exhibit reduced lipid absorption and resistance to diet-induced obesity following a HFD. This phenotype is reversed upon colonization with microbes harvested from the SI of mice fed a HFD and irrespective of the diet consumed thereafter.^106^ Furthermore, proximal SI epithelial organoids grown in media conditioned by SI *Clostridium bifermentans* cultures show increased expression of lipid absorption genes such as *Dgat2*, suggesting that SI bacteria play a key role in facilitating digestive and absorptive responses in the SI to dietary lipids, important for host lipid regulation. Additional evidence linking microbes residing in the SI to hyperlipidemia involve bile acid (BA) metabolism.^107^ As detailed earlier, BAs are important components of lipid digestion and absorption,^56^ and regulation of BA pools is not only critical for their function but also contributes to the management of total blood serum cholesterol levels.^107^ BA synthesis is under the control of specific hepatic enzymes, depending on the pathway: classic (CYP7A1 & CYP8B1) or alternative (CYP27A1 & CYP7B1). Hepatic expression of BA-synthesizing enzymes is regulated by nuclear receptor FXR activity in the ileum, which mediates its effects through a fibroblast growth factor (FGF15)-dependent pathway.^55^ Increased FXR-FGF15 signaling leads to suppressed hepatic BA-synthesizing enzyme expression and thus reduced BA synthesis. Ileal FXR activity is dependent on the binding of specific BAs. Unconjugated BAs tend to increase ileal FXR activity, whilst conjugated BAs are mainly FXR antagonists or weak agonists.^55,108^ The proportion of conjugated to unconjugated BAs in the SI is modulated by resident bacteria - *Lactobacillus, Bacillus, Streptococcus*, and *Lactococcus* - which, via intrinsic bile salt hydrolase (BSH) activity, deconjugate conjugated BAs.^55,109,110^ The SI microbiota can therefore inhibit hepatic BA synthesis – and in return increase serum cholesterol levels – through BA pool modulation resulting in increased FXR-FGF15 signaling. This is further demonstrated in the study of Huang et al. investigating the lipid-reducing effects of a famous traditional Chinese tea, Pu-erh, using mice.^108^ Pu-erh tea, and more specifically its constituent theabrownin, reduces ileal abundances of BSH-producing microbes and attenuates ileal BSH activity, both *in vivo* and in cultured ileal microbes. This leads to tauro-conjugated BA accumulation in the ileum, FXR-FGF15 signaling inhibition and finally increased hepatic BA synthesis, with a subsequent reduction in serum and hepatic cholesterol and triglyceride levels (Figure 1f).

### Environmental enteric dysfunction

Environmental enteric dysfunction (EED) is a disease of the SI characterized histopathologically by diminution in the number and height of intestinal villi, disruption of the epithelial barrier, and chronic inflammatory infiltrate. EED is a prevalent health problem, especially in developing countries. It is clinically associated with malabsorption and diarrhea and is thought to play a role in undernutrition, possibly mediated through the intestinal microbiota. Investigating this hypothesis using a collection of duodenal aspirate samples from 80 children with biopsy-confirmed EED, Chen and colleagues identified a strong correlation between the absolute levels of a group of 14 duodenal bacteria – among others *Veillonella sp*., *Streptococcus sp*., and *Rothia mucilaginosa* - and the degree of stunting.^111^ Furthermore, gnotobiotic mice colonized with duodenal strains cultured from children with EED developed enteropathy of the SI. Taken together, these results suggest a causal relationship between the SI microbiota, EED development, and growth stunting. Therapies targeting these EED-associated microbial changes may therefore prove vital for fighting undernutrition.

### Liver disease (cirrhosis)

Cirrhosis describes scarring (fibrosis) of the liver as a result of long-term damage to the liver. Patients with cirrhosis, particularly when decompensated, have increased risk of developing a range of serious complications including systemic infections, spontaneous bacterial peritonitis, hepatic encephalopathy, and acute-on-chronic liver failure.^112,113^ Underlying this increased risk is thought to be increased intestinal permeability, specifically in the duodenum. ^114^ Investigating the relationship between mucosal bacteria and epithelial permeability in patients with compensated cirrhosis, Bloom et al., identified a distinct duodenal microbial community in the duodenum, characterized predominantly by increased *Pseudomonadaceae* (*Proteobacteria)* and decreased *Lactobacillus*, *Bifidobacterium*, and *Clostridia* species, that was associated with increased epithelial permeability.^115^ Although further studies are required to establish underlying mechanisms, previous reports have shown specific *Lactobacillus* and *Bifidobacterium* strains to decrease intestinal permeability,^116,117^ suggesting a role for the mucosa-associated duodenal microbiota in the development of complications associated with cirrhosis.

### Inflammatory/Autoimmune disorders

A breakdown in the ability of the intestinal immune system to maintain a balance between immunity and tolerance underlies the development of several inflammatory-mediated disorders. As discussed previously, SI bacteria play an important role in this regulation. Consistent with this, studies have implicated the SI microbiota in a number of inflammatory disorders, with the underlying mechanisms slowly being unraveled. Here, we will focus on autoimmune/inflammatory diseases: IBD, pouchitis, type 1 diabetes and celiac disease.

### Inflammatory bowel disease

IBD is a chronic relapsing and remitting inflammatory disorder of the GI tract, with the two most common clinical manifestations being Crohn’s disease (CD) and ulcerative colitis (UC). IBD has been extensively studied in the context of the gut microbiota^118^ and several bacteria common to the SI, such as *Streptococci, Enterococci, Actinomyces, Veillonella* spp., and *Klebsiella pneumoniae* have been repeatedly implicated in its pathogenesis.^119–122^ Colonization of the common SI resident *Enterococcus faecalis* was also shown to induce IBD in genetically susceptible mice.^123^ These observations have been linked to the idea of oralization – the ectopic colonization of typically oral bacteria in the colon – which is hypothesized to exacerbate an already dysbiotic and inflammatory state in patients with IBD.^124^ This is demonstrated in the study of Atarashi et al., whereby ectopic intestinal colonization of orally derived *Klebsiella* isolates induced T_H_1 cell activation and subsequent intestinal inflammation in genetically susceptible individuals.^125^ However, given that *Klebsiella*, and other common oral/IBD-associated bacteria, are abundant in the SI, as well as the close proximity of the SI to the colon, one could hypothesize that in fact the SI serves as a reservoir for pathobionts to translocate to the colon and cause damage. Furthermore, diurnal regulation of the diet-microbiota-MHC class II-IL10 axis is critical for maintaining SI immune response and barrier integrity. Using a Crohn-like enteritis mouse model, Tuganbaev and colleagues showed that concomitant disruption of the MHC class II-IL10 axis further exacerbates intestinal inflammation.^88^ These findings suggest that perturbations in the SI microbiota contribute to IBD pathogenesis, in part, through dysregulation of an MHC class II-dependent IL-10 signaling pathway.

Differences in disease manifestation between CD and UC may also be explained by SI bacteria. Using a cohort of 359 treatment-naive pediatric patients with CD or UC, as well as control individuals, Haberman and colleagues were able to identify ileal gene expression profiles and microbial communities specific to CD, irrespective of disease location (i.e., ileum or colon).^126^ These results hint at a central role for the ileum, and its microbiota, in the induction of IBD subtype CD. The association between intracellular pattern recognition receptor, NOD2, and CD supports this further. NOD2 is highly expressed in ileal Paneth cells where it regulates the ileal microbiota through secretion of antimicrobial molecules and controls pro-inflammatory immune responses to intestinal microbiota through upregulation of anti-inflammatory mediators and downregulation of pro-inflammatory cytokines.^127,128^ Mutations in NOD2 are the most strongly associated genetic risk factors for ileal CD,^129–131^ suggesting that dysregulation of the SI microbiota contributes to CD disease pathogenesis. Increased consumption of emulsifiers is also strongly linked to an increased risk of CD.^132^ Dietary emulsifiers polysorbate-80 and carboxymethyl cellulose were reported to induce compositional changes in the ileal microbiota of mice that lead to exacerbation of indomethacin-induced SI lesions via an interleukin-1β signaling pathway.^133^ Finally, extra-intestinal manifestation of creeping fat in individuals with CD is another IBD subtype-distinguishing feature that has recently been attributed to small intestinal bacteria.^134^ Creeping fat results from the migration of mesenteric adipose tissue to inflamed regions in the intestine, primarily in the SI whereby it undergoes hyperplasia and wraps around the intestinal wall. Ha and colleagues demonstrated, using CD ileal surgical resections containing creeping fat and gnotobiotic mice, that this restructuring of mesenteric adipose tissue is promoted by the translocation of a collection of ileal mucosa-associated bacteria, including *Clostridium innocuum*.^134^

UC patients, particularly those with right-sided presentation, are also at risk of developing primary sclerosing cholangitis (PCS), whereby the colitis is thought to be the first manifestation of the disease. PSC is characterized by inflammation and progressive fibrosis of the bile ducts with eventually liver disease.(135) Microbial dysbiosis in the upper GI and bile duct fluid, including an increase in abundance of Veillonella dispar and E. coli at the duodenal mucosa has been reported.(136)

### Pouchitis

Although several different drug therapies are available to reduce symptoms and disease activity in individuals with IBD, an unfortunate proportion end up requiring a total colonic resection with an ileal pouch-anal anastomosis (pouch). This same surgical procedure is also carried out as a preventative treatment for individuals with familial adenomatous polyposis (FAP) due to a significantly increased risk of developing colorectal cancer.^137^ Notably, approximately 50% of individuals with a pouch due IBD go on to develop pouchitis – inflammation of the pouch – whereas this very rarely occurs in patients with FAP.^138,139^ Studies investigating the pathogenesis of pouchitis have found differences in pouch microbiomes between IBD and FAP pouches, as well as between UC pouches with and without pouchitis, indicating a role for the microbiota in driving pouchitis.^140,141^ This is further supported by the fact that pouchitis is often responsive to antibiotic treatment.^142^ In general, the microbiota of IBD pouches display a stronger shift toward a more colon-like composition, typified by a decrease in facultative anaerobes and an increase in obligate anaerobes, sulfate-reducing bacteria, and *Clostridia* species.^143^ In the study conducted by Sinha et al., metabolomic, transcriptomic, and metagenomic profiling in UC vs FAP pouches showed significantly reduced levels of SBAs, genes converting PBAs to SBAs and the family of SBA-producing bacteria, Ruminococcaceae^141^. Furthermore, SBA supplementation in mouse colitis models minimized the intestinal inflammation, in part via a TGR5 bile acid receptor-dependent pathway, suggesting that a previously existing dysbiosis in the newly created pouches of UC patients leads to SBA deficiency, which in turn drives a pro-inflammatory state.

### Type 1 diabetes

The body’s progressive self-destruction of the insulin producing beta cells in the pancreas, causing type 1 diabetes (T1D), is increasingly being linked to intestinal abnormalities.^144^ These alterations include dysfunctional epithelial barrier function and abnormally active immune system. The role of the gut microbiota has therefore gained attention in this respect, including the SI microbiota given its close functional and spatial relationship with the pancreas, as well as shared blood supply. By means of inflammatory profiling and microbiome evaluation of duodenal biopsies from patients with T1D (*n* = 19), patients with celiac disease (included as controls for intestinal inflammatory disease; *n* = 19) and healthy controls (*n* = 16), Pellegrini et al., identified a T1D-specific microbial signature in the duodenal mucosa that correlated with a pro-inflammatory gene expression profile.^144^ The distinct microbial signature was characterized by an increase in Firmicutes and Firmicutes/Bacteroidetes ratio and a reduction in Proteobacteria and Bacteroidetes. Similar changes were also observed in the duodenal microbiota of diabetic rats.^145^ Notably, despite an overall increase in Firmicutes in duodenal mucosal biopsies from patients with T1D as compared to the healthy controls, *Clostridia* species abundances were reduced. *Clostridia* contribute significantly to butyrate production in the gut and are also key mucin-degrading bacteria, both of which are important for barrier integrity and possibly preventing autoantibody formation.^146,147^ Segmented filamentous bacterial strains in non-obese diabetic mice were also shown to induce autoimmune diabetes via T-helper 17 cell interaction in the SI lamina propria.^148^ Further evidence implicating the SI microbiota in T1D pathogenesis comes from the fecal microbiota transplantation (FMT) study of de Groot et al. in 2021.^149^ FMT preserved the residual beta cell function, halting the decline of endogenous insulin production. The mechanisms of which were linked to changes in both fecal and SI microbiota composition, SI gene expression, metabolite profiles, and T cell immunity.

### Celiac disease

Celiac disease (CeD) is characterized by an intolerance for gluten, aberrantly activating the body’s immune system and causing damage, predominantly, to the SI.^150^ This loss of tolerance for gluten has been hypothesized to involve increased mucosal permeability and subsequent recruitment of T cells, brought about by changes in the intestinal microbial composition.^151^ Analysis of duodenal biopsies from patients with CeD have revealed a dominance of *Proteobacteria* such as *Enterobacteriaceae* bacteria, as well as a high prevalence of *Bacteroidetes* and *Streptococcus* species.^151,152^ *Pseudomonas aeruginosa* abundances are also increased in the duodenum of patients with CeD.^153^ *P. aeruginosa* is able to metabolize gluten, producing immunogenic peptides that more efficiently permeate the gut barrier and activate gluten-specific T cells. The bacteria also express a specific elastase that synergizes with gluten to amplify inflammation. Potential metabolic changes associated with the microbial changes in the duodenum of patients with CeD include an increase in alternative pathways for energy production such as D-glucarate, L-arabinose, D-galactarate, and biogenic amine degradation pathways and decreased SCFA production.^152^ A gluten-free diet is currently the only available and effective treatment for CeD.^150^ Complete remission, however, is not guaranteed, with some individuals still experiencing symptoms despite adhering to the strict diet. The role of duodenal microbiota in the persistence of symptoms following the removal of gluten from the diet was investigated by a group in Sweden.^154^ Patients on a gluten-free diet suffering from persistent symptoms, compared to without symptoms, exhibited lower microbial richness, higher relative abundance of Proteobacteria, and lower relative abundance of Bacteroidetes and Firmicutes. Although in its infancy, these results provide insight into possible future treatment alternatives for CeD patients.

### Neurological disorders

The gut–brain axis describes the bidirectional interaction between the gut and brain that influences both neurological and intestinal functions.^155^ Increasingly more research is being conducted on the role played by gut microbes in effecting this two-way relationship, and a couple of studies focus specifically on the SI.

Already inside the womb, the influence of the SI microbiota on the brain is significant. Mice born to mothers exposed to a synthetic double-stranded RNA that mimics viral infection during pregnancy – eliciting an immune response termed maternal immune activation (MIA) – display cortical brain lesions and abnormal behavioral phenotypes.^156^ In contrast, offspring from antibiotic treated pregnant mice do not exhibit such abnormal phenotypes, suggesting that maternal intestinal microbes are required for the induction of MIA-associated behavioral and brain abnormalities in offspring. Ileal mucosa-associated segmented filamentous bacteria in pregnant mice are necessary to induce these neurodevelopmental disorders via T_H_17 cell expansion and activation.

Exploring the contribution of psychological stress toward disease exacerbation in patients with CD (i.e., increased intestinal inflammation), Shaler and colleagues observed a number of changes in the ileum of conventional specific-pathogen-free mice subjected to acute psychological stress.^157^ Firstly, psychological stress induced ileal nutritional immunity, evident by an upregulation of host genes associated with nutritional immunity and metal restriction, as well as glucocorticoid-mediated depletion of IL-22-producing immune cells that activate the protective mucosal antimicrobial defenses. This in turn led to ileal dysbiosis dominated by an expansion of, in particular, adherent-invasive *Escherichia coli* (AIEC) - a gut pathobiont that can evade SI defense mechanisms and is known to colonize ileal lesions in patients with CD.^157,158^ Deeper analysis showed that AIEC takes advantage of the increased nutritional immunity, in part through the expression of iron-scavenging siderophores, as well as the impaired host immunity, permitting its expansion and further enhancing the proinflammatory state in patients with CD. Other genera commonly found enriched in CD patients such as *Enterococcus faecalis* were also enriched following exposure to physiological stress, although this was not further explored. The results highlight the complex role of the SI microbiota within the gut–brain axis to influence intestinal health, which warrants further investigation.

The study of Miyauchi et al. highlights the potential of the SI microbiota to drive neurological disease, namely multiple sclerosis (MS).^159^ MS is an autoimmune-driven demyelinating disease of the brain and spinal cord. Miyauchi and colleagues demonstrated that mice treated orally with ampicillin were protected against demyelination of the spinal cord and infiltration of the spinal cord by inflammatory cells, hinting at a gut microbiota-mediated inflammatory response underlying MS.^159^ Further investigation using GF mice colonized with specific ampicillin-sensitive SI bacteria revealed that SI microorganisms act synergistically to activate myelin oligodendrocyte glycoprotein-specific T cells. These T cells are likely responsible for the pro-inflammatory and demyelination phenotype of experimental autoimmune encephalomyelitis mice. Given these results and previous reports of increased T_H_17 cells in the SI of patients with MS,^160^ future efforts should be made to explore the human SI microbiota in the context of MS.

## Conclusion and future perspectives

The SI plays an essential role in host metabolism, immunity, and endocrine and neurological functions. Despite this, and the growing interest in its microbial communities, knowledge of the SI microbiota is still in its infancy and greatly lags behind that of the lower intestinal tract. In this review, we detailed the current methods used to sample and model the SI microbiota and their limitations. We also discussed the function of the SI in host metabolism and immunity and summarized the studies that provide evidence for, and insight into, the role played by the SI microbiota, including in the context of disease.

The SI microbiota is emerging as a critical component of human health by regulating fundamental processes in the SI that not only impact intestinal health but also influence extraintestinal physiological functions. In recent years, there has been a surge in fecal microbiota transplantation studies and a drive to develop clinical interventions, but with limited success. A potential reason for this could be the current bias toward fecal microbiome studies, upon which these trials are based, ignoring the important contribution of the SI microbiota. A better understanding of the metabolic capacity of the SI microbiota and its interactions with the host therefore has the potential to significantly advance gut microbiome research and its quest for novel diagnostic techniques and intervention strategies to improve human health. Similarly, dietary interventions have been designed following the conclusions derived from fecal microbiome studies. Considering that the small intestine is the main organ involved in nutrient absorption and metabolic regulation, future interventions should consider host–microbiota interactions within this organ.

One of the major factors restricting SI microbiota research, however, are the limitations and challenges of the current methods for sampling and modeling this poorly accessible and highly dynamic ecosystem. The field would therefore greatly benefit from more effort being made to develop uniform sampling techniques that provide accurate representations of the SI microbiota, are less invasive, and do not require specialists to perform. Additionally, the majority of our knowledge on the mechanisms underlying the SI microbiota–host interactions is derived from mouse/animal models. Developing *in vitro* or in *vivo* models that better recapitulate the human SI environment will be paramount to translating promising findings into human intervention studies and clinical trials.

## Data Availability

Data sharing is not applicable to this article as no new data were created or analyzed in this review.
